# Inter-phantom variability in digital mammography: implications for quality control

**DOI:** 10.1186/s41747-025-00583-0

**Published:** 2025-04-26

**Authors:** Gisella Gennaro, Gilberto Contento, Andrea Ballaminut, Francesca Caumo

**Affiliations:** 1 Veneto Institute of Oncology (IOV), IRCCS, Padua, Italy; 2 Cyberqual srl, Gorizia, Italy

**Keywords:** Data accuracy, Mammography, Phantoms (imaging), Quality control, Reproducibility of results

## Abstract

**Background:**

Phantoms play a critical role in mammography quality control (QC) by providing standardized conditions for evaluating image quality (IQ) metrics. However, inter-phantom variability may affect the reliability of these metrics, especially for inter-system comparisons. The aim of this study was to quantify the intra- and inter-phantom variability of IQ metrics using a set of theoretically identical phantoms.

**Methods:**

Twenty-four TORMAS phantoms were imaged ten times each using a mammography unit under standardized high-dose conditions. Images were analyzed using automated software to extract 64 IQ metrics, including contrast-to-noise ratio (CNR) as well as modulation transfer function (MTF)-related and other metrics. Outliers were identified and excluded. Variability was assessed by calculating intra- and inter-phantom variances and coefficients of variation (COVs). The relative contributions of intra- and inter-phantom variability to total variability were also determined.

**Results:**

Two defective phantoms were excluded. Analysis of 64 IQ metrics across 22 phantoms showed higher inter-phantom variability compared to intra-phantom variability. Mean intra- and inter-phantom COVs were 6.9% and 15.1% for the 34 CNR metrics, 4.8% and 5.4% for the 5 MTF-related metrics, 0.14% and 0.75% for the 10 contrast metrics, 4.9% and 14.8% for the 15 noise metrics, respectively. Inter-phantom variability contributed 84.2% to total variability, highlighting its dominance.

**Conclusion:**

Inter-phantom variability significantly affects IQ metrics, emphasizing the importance of using the same phantom for inter-system comparisons to avoid confounding results. Conversely, phantoms are well-suited for assessing system reproducibility over time, focus on inter-system variability while consistently using a single phantom.

**Relevance statement:**

This study highlights the significant impact of inter-phantom variability on image quality assessment, emphasizing the importance of using the same phantom for benchmarking imaging systems. These findings are crucial for optimizing quality control protocols and ensuring reliable, reproducible evaluations.

**Key Points:**

- Inter-phantom variability exceeded intra-phantom variability across all image quality metrics of digital mammography.
- Subtle details showed higher total variability compared to more distinct features.
- Modulation transfer function metrics exhibited comparable intra- and inter-phantom variability, highlighting positioning sensitivity.
- Inter-phantom variability contributes 84% to total variability, impacting imaging system comparisons.
- Using the same phantom ensures reliability in imaging system performance evaluations.

**Graphical Abstract:**

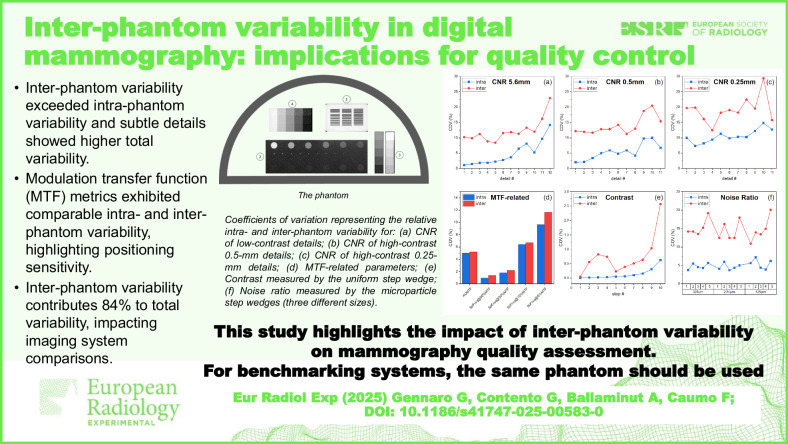

## Background

In medical imaging, the term “phantoms” or test objects denotes physical models designed to mimic specific characteristics of human tissues or organs. These phantoms serve as standardized tools for testing, calibrating, and optimizing imaging systems and techniques without involving human subjects [1]. In mammography, the primary method for early detection of breast cancer and population-based screening programs, it is imperative that imaging systems effectively capture subtle changes and abnormalities, such as microcalcifications or masses, against the background of normal breast tissue. To ensure accurate detection of these features, mammography phantoms for quality control (QC) were introduced from the beginning [1–3]. Typically constructed from materials such as polymers, plastics, and epoxy resins, mammography phantoms are designed to mimic the attenuation properties of breast tissue within the mammography-specific X-ray energy spectrum [4]. These phantoms can embed a variable number of details to measure one or more image quality (IQ) metrics. Usually, the details reproduce breast lesions such as microcalcifications or low-contrast masses and may incorporate contrast objects such as stepwedges, as well as spatial resolution patterns [1, 3, 5]. Phantom images can be assessed either by visual evaluation or by quantitative measures [6] provided by more or less automated software tools [7–11].

The general assumption in phantom-based studies is that two or more phantoms of the same type, under the same exposure conditions, produce consistent results regardless of the method used to assess image quality. Under this assumption, some phantoms are used for acceptance and commissioning tests, where the goal is to determine whether a mammography unit meets the minimum performance requirements for clinical use [12, 13]. However, variability can arise from two key sources: intra-phantom and inter-phantom variability. Intra-phantom variability, reflecting fluctuations in repeated measurements of the same phantom under identical conditions, is influenced by factors such as imaging system reproducibility and phantom repositioning. It serves as a useful surrogate for assessing imaging equipment consistency and is routinely assessed in phantom studies. Inter-phantom variability, on the other hand, arises from differences between phantoms of the same type, including variations in manufacturing, material properties, and structural alignment. These differences can significantly impact the reliability of IQ metrics, particularly in studies comparing different imaging systems or testing protocols. Despite its potential implication for QC, inter-phantom variability remains underexplored in the literature, leaving a gap in understanding its role in mammography QC processes [14–17].

To compound the problem, phantom manufacturers often provide limited or vague specifications, lacking detailed tolerances for the parameters influencing the physical characteristics of the phantom and its embedded details. This lack of standardization complicates efforts to ensure consistency among phantoms, further underscoring the need for comprehensive studies that address inter-phantom variability and its implications.

In this study, we investigated inter-phantom variability using 24 phantoms of the same brand and model that were inspected for defects and evaluated under standardized conditions. These phantoms were part of a regional research initiative to harmonize QC procedures across 24 mammography screening sites [18]. By analyzing intra- and inter-phantom variability across 64 IQ metrics, this study aims to provide critical insight into the role of phantom variability in mammography QC, with practical recommendations for users.

## Methods

### Phantoms

The TORMAS phantom (Leeds Test Objects Ltd., Leeds, UK) includes a semicircular test plate (22 cm in diameter) designed to simulate the absorption characteristics of breast tissue when used with an attenuator stack of acrylic plates. The test plate features are the following:

- a high-contrast resolution grating with 26 bar patterns spanning spatial frequencies from 1 to 20 lp/mm for spatial resolution evaluation;
- low-contrast details (12 circular details, 5.6 mm in diameter).
- two sets of high-contrast details (11 circular details each, 0.5 mm and 0.25 mm in diameter).
- a step wedge with 8-mm square strips creating a ten-point gray scale;
- 3 step wedges with irregularly shaped particles, with median sizes of 125, 234, and 328 µm.

**Fig. 1:**
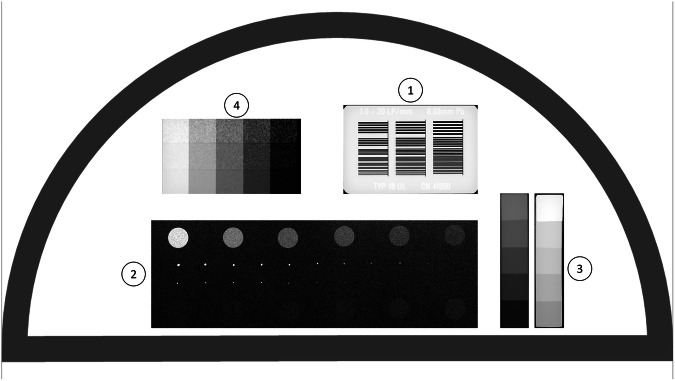
Scheme of the TORMAS phantom (Leeds Test Object Ltd.), consisting of a semicircular test plate embedding four different groups of details: (1) a high-contrast resolution gratings for spatial resolution measurements; (2) a contrast-detail part, including 12 low-contrast 5.6-mm details, 11 high-contrast 0.5-mm, and 11 high-contrast 0.25-mm details; (3) a step wedge with 10 steps of different contrast; (4) three step wedges 5-step each with microparticles with median size 125, 234, and 328 µm for noise measurements

A schematic of the TORMAS phantom is depicted in Fig. 1.

The attenuator stack consists of six 10-mm plates and two 5-mm plates, allowing for customizable phantom thickness. Routine QC is typically performed using the TORMAS with a 35-mm stack of acrylic plates, yielding attenuation equivalent to a 45-mm-thick breast [19].

### Phantom testing

Twenty-four TORMAS phantoms were tested on the same mammography unit before being distributed to 24 screening sites for automated routine QC testing [18]. The test plate, which contains the details/objects used to assess image quality, was the primary focus of the study. In contrast, the attenuator stack, which simulates the typical absorption of breast tissue, may reduce the visibility of these details in the resulting images. As a result, the experiment was designed to evaluate inter-phantom variability using only the 10-mm thick test plates.

The addition of the standard 35-mm polymethyl methacrylate stack, while consistent with routine clinical QC protocols, would have resulted in significant attenuation, compromising the detectability of many low-contrast and small details. As a result, the number of measurable metrics would have been reduced, and the values of those that remained detectable would have been diminished. By choosing the 10-mm test plate, we prioritized maximizing the visibility of all embedded details to ensure that a comprehensive set of IQ metrics could be evaluated.

Ten images of each phantom test plate were acquired using a Senographe DS system (GE Healthcare, Chalfont St. Giles, Buckinghamshire, UK), exposing the TORMAS phantoms in manual exposure mode using Mo/Mo, 28 kVp and 50 mAs. At each exposure, each phantom was manually repositioned. The test plate, being semicircular, was visually aligned with the chest wall side of the breast support on the mammography unit. Since no alignment marks were present, the linear side of the test plate was adjusted by sight to approximate the same placement for each acquisition. The exposure mode being manual, no compression was required; however, the compression paddle was positioned close to the phantom surface to replicate scatter conditions similar to those in automatic exposure mode. This setup also facilitated phantom repositioning for evaluating intra-phantom variability. This manual process introduced small translations relative to the ‘central’ point of the phantom or minor rotational deviations, which were intentionally included to simulate real-world conditions. These variations ensured that all factors contributing to intra-phantom variability were captured in the analysis.

All unprocessed images (“DICOM For Processing”) were processed by Automatic Phantom Image Analysis (AutoPIA) version 3.7.14 (CyberQual s.r.l., Gorizia, Italy), an advanced automated software designed to accurately recognize the objects embedded in the phantom and dynamically locate regions of interest (ROIs) for the measurement of a large number of IQ metrics. Unlike methods that rely on preset ROIs, AutoPIA automatically adjusts ROI placement based on the actual position and orientation of the phantom details in each image, ensuring accurate measurements regardless of minor translations or rotations introduced during phantom positioning. IQ metrics derived by these objects include the contrast-to-noise ratio (CNR) of low-contrast 5.6-mm and high-contrast 0.5-mm and 0.25-mm details, the area under the modulation transfer function (AUMTF), the spatial frequencies corresponding to 50%, 20%, 10%, 5% of the MTF, the relative contrast of the 10 steps of the uniform step wedge, and the variance ratio between the 15 steps of the step wedges incorporating microparticles and the uniform step wedge, used as a metric to assess structured over quantum noise. The IQ metrics measured by AutoPIA have been previously described [18] and are summarized in Table 1.

**Table 1:** Definition of the image quality metrics measured by the automated software AutoPIA from the TORMAS phantom images

Metric type	Metric name	Metric description	Number of metrics
Contrast-to-noise ratio	CNR5.6 mm	CNR of low-contrast details of 5.6 mm: It is calculated as the difference in signal between the detail and the surrounding background (contrast) divided by the background noise. This is achieved by placing two circular regions of interest (ROIs): one inside the detail and the other in the surrounding background. The mean and standard deviation of pixel values within these ROIs are used to compute the CNR. The CNR for each detail is affected by all dose-related factors, including tube voltage, filtration, and mAs.	12
	CNR0.5 mm	CNR of high-contrast details of 0.5 mm: Same definition as the CNR of large details. For small details, estimating the detail signal requires a more sophisticated algorithm that takes into account the signal strength on a pixel-by-pixel basis and weights them appropriately. This approach compensates for the inability to define a regular ROI as used for larger objects. The CNR of small details is affected by all dose-related factors, including tube voltage, filtration, and mAs.	11
	CNR0.25 mm	CNR of high-contrast details of 0.25 mm: Same definition as the CNR of large details. For small details, estimating the detail signal requires a more sophisticated algorithm that takes into account the signal strength on a pixel-by-pixel basis and weights them appropriately. This approach compensates for the inability to define a regular ROI as used for larger objects. The CNR of small details is affected by all dose-related factors, including tube voltage, filtration, and mAs.	11
MTF-related	AUMTF	Area under the modulation transfer function curve (AUMTF): This is a quantitative measure of the overall spatial resolution of an imaging system. It is obtained by integrating the modulation transfer function (MTF) over a range of spatial frequencies. The MTF, which represents the system’s ability to reproduce contrast at different spatial resolutions, is measured using a resolution pattern. Specifically, the method by Droege and Morin is used, in which the MTF is calculated from the intensity profile of the bar patterns by determining the modulation depth of the bars imaged at different spatial frequencies.	1
	SpFreq@50%MTF	Spatial frequency at 50% of MTF	1
	SpFreq@20%MTF	Spatial frequency at 20% of MTF	1
	SpFreq@10%MTF	Spatial frequency at 10% of MTF	1
	SpFreq@5%MTF	Spatial frequency at 5% of MTF	1
Contrast	Contrast	Contrast is defined as the relative difference in signal intensity, expressed as a percentage, between a given step of a stepwedge (with 10 steps) and the adjacent background. It quantifies the ability of the imaging system to distinguish signal variations and depends on technical factors such as tube voltage and filtration, which shape the X-ray spectrum.	10
Noise ratio	NR	The noise ratio is a metric calculated for the three stepwedges, each containing 5 steps and covered with microparticles of median sizes 328, 234, and 125 µm. For each step, the noise ratio is defined as the ratio of the variance of pixel values within the step to the variance of pixel values within the “corresponding step” (same contrast level) of the uniform stepwedge used for contrast measurements. This parameter provides insight into how the structured noise (introduced by microparticles) compares with the quantum noise of a uniform background.	15

*AUMTF* Area under the MTF curve, *MTF* Modulation transfer function, *ROIs* Regions of interest, *SpFreq* Spatial frequency

### Statistical analyses

The first step was to identify potential “defective” phantoms. For each metric, the overall mean (also referred to as “grand mean”) was calculated and used to detect outliers among the phantoms. Phantoms with mean measurements that deviated by more than ± 3 standard deviations from the grand mean were considered outliers and excluded from further analysis.

Once the defective phantoms were removed, intra-phantom variability was calculated for each remaining phantom and metric by computing the sample variance (the squared standard deviation) of the 10 measurements obtained from the repeated images for each phantom. This intra-phantom variability was denoted as ${\sigma }_{{intra}}^{2}$.

The mean value of the 10 repeated measurements for each phantom was then calculated for each metric, and these mean values were used to compute the inter-phantom variability, denoted as ${\sigma }_{{inter}}^{2}$, which reflects the variance across all remaining phantoms.

The total variability was determined as the sum of intra- and inter-phantom variabilities,

$${\sigma }^{2}={\sigma }_{{intra}}^{2}+{\sigma }_{{inter}}^{2}$$

The relative contributions of intra- and inter-phantom variability to the total variability were calculated as percentages using the following formulas:

$${Intra}-{phantom\; variability}( \% )=\left(\frac{{\sigma }_{{intra}}^{2}}{{\sigma }^{2}}\right)\times 100$$

$${Inter}-{phantom\; variability}( \% )=\left(\frac{{\sigma }_{{inter}}^{2}}{{\sigma }^{2}}\right)\times 100$$

This approach provided a weighted assessment of the contributions of intra- and inter-phantom variability to the overall variability in the measured image quality metrics.

Variability was also calculated using coefficients of variation (COVs), defined as the ratio between the standard deviation and the mean, expressed as a percentage. The intra-phantom COV quantified the relative variability of repeated measurements within each phantom, while the inter-phantom COV measured the relative variability of the average measurements across all phantoms.

In addition, the small variability of the equipment was verified by calculating the variance of the entrance dose obtained from the appropriate DICOM tag.

Statistical analysis was performed by Google Sheets and by OriginPro 2020b (OriginLab Corporation, Northampton, MA, USA) using standard functions/modules. The dataset analyzed during the current study is available in the Zenodo repository at 10.5281/zenodo.14363482.

## Results

**Fig. 2:**
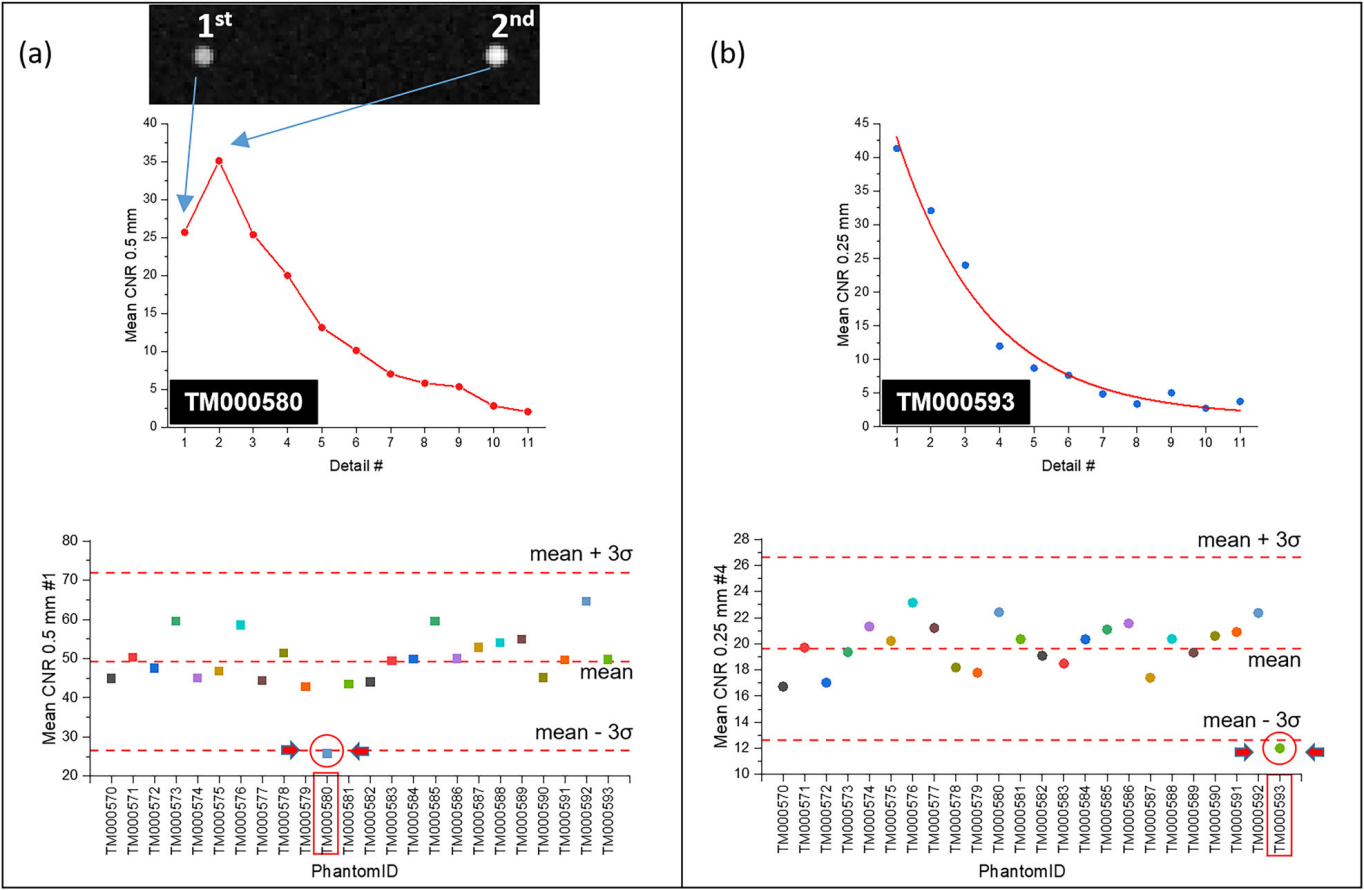
Identification of two defective phantoms. **a** Phantom TM000580 showed an abnormally low CNR value for the first high-contrast detail of 0.5 mm, which was paradoxically lower than the CNR of the second detail. This anomaly was likely caused by an exchange of the two details, as shown in the phantom image snippet. **b** Phantom TM000593 showed an abnormally low CNR value for the fourth high-contrast detail of 0.25 mm. These two phantoms were identified as outliers and excluded from further analysis. CNR, Contrast-to-noise ratio

The mean CNR, averaged across 10 repeated images, for the 0.5-mm details of phantom TM000580 along with the distribution of CNR_0.50mm#1_ values across all phantoms are shown in Fig. 2a. Similarly, the mean CNR for the 0.25-mm details of phantom TM000593 and the distribution of CNR_0.25mm#4_ values for all phantoms are shown in Fig. 2b. Both CNR_0.50mm#1_ and CNR_0.25mm#4_ were identified as outliers for their respective phantoms, leading to the exclusion of these two defective phantoms from further analysis.

Intra- and inter-phantom variability, expressed as variances, were calculated for the remaining 22 phantoms across all 64 metrics, as shown in Table 2. Results consistently indicated that inter-phantom variability exceeded intra-phantom variability for every metric analyzed.

**Table 2:** Intra-phantom, inter-phantom, and total variability, expressed as variance, for all 64 image quality metrics evaluated in this study

Metric	Intra-phantom variability (${{\sigma }}_{{intra}}^{\bf 2}$)	Inter-phantom variability (${{\sigma }}_{{inter}}^{\bf 2}$)	Total variability (${{\sigma }_{{intra}}^{\bf 2}+\sigma }_{{inter}}^{\bf 2}$)	Total* (mean ± standard deviation)
CNR5.6 mm#1 CNR5.6 mm#2 CNR5.6 mm#3 CNR5.6 mm#4 CNR5.6 mm#5 CNR5.6 mm#6 CNR5.6 mm#7 CNR5.6 mm#8 CNR5.6 mm#9 CNR5.6 mm#10 CNR5.6 mm#11 CNR5.6 mm#12	0.0260 0.0183 0.0173 0.0076 0.0051 0.0032 0.0021 0.0050 0.0028 0.0009 0.0013 0.0014	2.0114 0.8355 0.6246 0.1665 0.0657 0.0510 0.0208 0.0139 0.0072 0.0042 0.0033 0.0034	2.0374 0.8539 0.6419 0.1741 0.0709 0.0541 0.0229 0.0188 0.0100 0.0051 0.0046 0.0049	13.88 ± 1.43 9.24 ± 0.92 7.04 ± 0.80 4.61 ± 0.42 3.04 ± 0.27 1.95 ± 0.23 1.22 ± 0.15 1.04 ± 0.14 0.64 ± 0.10 0.54 ± 0.07 0.35 ± 0.07 0.26 ± 0.07
CNR0.5 mm#1 CNR0.5 mm#2 CNR0.5 mm#3 CNR0.5 mm#4 CNR0.5 mm#5 CNR0.5 mm#6 CNR0.5 mm#7 CNR0.5 mm#8 CNR0.5 mm#9 CNR0.5 mm#10 CNR0.5 mm#11	1.1331 0.6609 0.9087 1.1523 0.7991 0.2759 0.1860 0.0552 0.2690 0.0789 0.0158	37.6681 20.0780 9.9423 7.2977 3.4523 2.2621 0.6492 0.4865 0.9088 0.2874 0.0715	38.8012 20.7389 10.8510 8.4500 4.2515 2.5380 0.8353 0.5417 1.1777 0.3663 0.0873	50.34 ± 6.23 37.40 ± 4.55 26.97 ± 3.29 21.07 ± 2.91 14.48 ± 2.06 10.57 ± 1.59 7.13 ± 0.91 5.38 ± 0.74 5.10 ± 1.09 2.62 ± 0.61 1.74 ± 0.30
CNR0.25 mm#1 CNR0.25 mm#2 CNR0.25 mm#3 CNR0.25 mm#4 CNR0.25 mm#5 CNR0.25 mm#6 CNR0.25 mm#7 CNR0.25 mm#8 CNR0.25 mm#9 CNR0.25 mm#10 CNR0.25 mm#11	19.2477 6.9268 4.2382 3.6529 2.6029 1.0759 0.5677 0.3251 0.2356 0.1847 0.0835	67.2653 45.6315 16.2410 6.1367 6.3777 3.8857 1.6656 1.3644 0.6010 0.6558 0.1232	86.5129 52.5583 20.4792 9.7896 8.9806 4.9616 2.2333 1.6895 0.8367 0.8405 0.2066	41.70 ± 9.30 34.12 ± 7.25 25.06 ± 4.53 19.84 ± 3.13 13.91 ± 3.00 10.38 ± 2.23 7.09 ± 1.49 5.21 ± 1.30 3.97 ± 0.91 2.76 ± 0.92 2.23 ± 0.45
AUMTF SpFr@50%MTF SpFr@20%MTF SpFr@10%MTF SpFr@5%MTF	0.0370 0.0016 0.0176 0.3366 1.0087	0.0375 0.0035 0.0247 0.3416 1.3615	0.0745 0.0051 0.0423 0.6782 2.3702	3.71 ± 0.27 4.25 ± 0.07 7.08 ± 0.21 8.70 ± 0.82 10.02 ± 1.54
Contrast#1 Contrast#2 Contrast#3 Contrast#4 Contrast#5 Contrast#6 Contrast#7 Contrast#8 Contrast#9 Contrast#10	0.0001 0.0002 0.0004 0.0007 0.0013 0.0013 0.0029 0.0045 0.0093 0.0098	0.0018 0.2347 0.4695 0.3328 0.0274 0.0529 0.0700 0.0729 0.1001 0.1587	0.0018 0.2349 0.4698 0.3335 0.0286 0.0542 0.0729 0.0774 0.1094 0.1685	(95.24 ± 0.04)% (86.86 ± 0.48)% (83.29 ± 0.69)% (77.84 ± 0.58)% (71.04 ± 0.17)% (59.08 ± 0.23)% (52.05 ± 0.27)% (42.51 ± 0.28)% (30.46 ± 0.33)% (15.50 ± 0.41)%
NR328µm#1 NR328µm#2 NR328µm#3 NR328µm#4 NR328µm#5 NR234µm#1 NR234µm#2 NR234µm#3 NR234µm#4 NR234µm#5 NR125µm#1 NR125µm#2 NR125µm#3 NR125µm#4 NR125µm#5	0.1103 2.9171 6.9220 15.231 68.0262 0.0227 0.5044 0.7032 2.3572 8.0035 0.0207 0.1252 0.1218 0.2113 1.3981	1.4564 18.1206 56.0662 188.1026 774.0175 0.1967 3.4391 7.1322 18.6005 99.3075 0.0724 0.4446 1.0879 2.9645 13.7880	1.5668 21.0376 62.9882 203.3334 842.0437 0.2195 3.9436 7.8355 20.9578 107.3110 0.0931 0.5698 1.2097 3.1758 15.1861	8.48 ± 1.25 29.92 ± 4.59 55.28 ± 7.94 89.98 ± 14.26 145.27 ± 29.02 3.54 ± 0.47 11.42 ± 1.99 21.38 ± 2.80 34.49 ± 4.58 55.61 ± 10.36 2.46 ± 0.31 4.78 ± 0.75 7.75 ± 1.10 11.51 ± 1.78 18.51 ± 3.90

*AUMTF* Area under the MTF curve, *CNR* Contrast-to-noise ratio, *MTF* Modulation transfer function, *NR* Noise ratio, *SpFreq* Spatial frequency

* The last column shows the overall mean and standard deviation calculated as the square root of the total variance for each metric

**Fig. 3:**
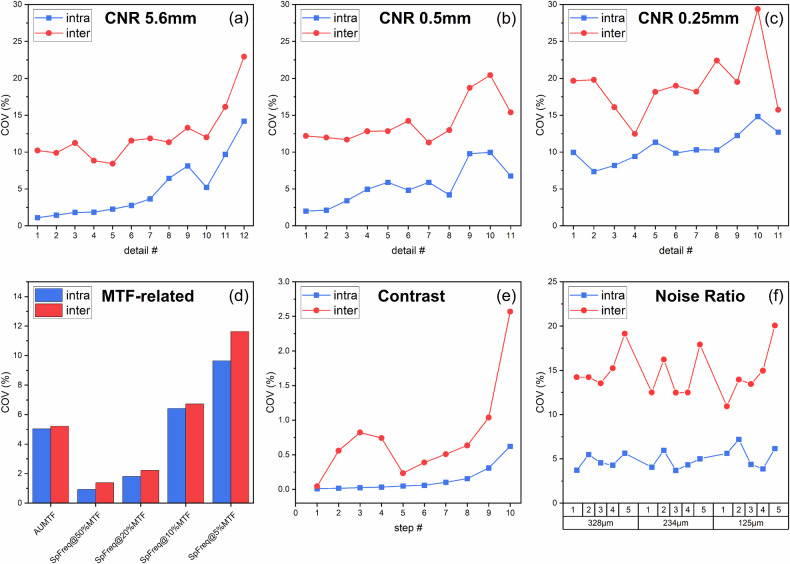
Coefficients of variation (COVs) representing the intra- and inter-phantom variability for all metrics evaluated in this study: **a** contrast-to-noise ratio (CNR) of low-contrast 5.6-mm details; **b** CNR of high-contrast 0.5-mm details; **c** CNR of high-contrast 0.25-mm details; **d** MTF-related parameters including the area under the modulation transfer function (MTF) curve (AUTF) and the spatial frequency corresponding to 50%, 20%, 10%, and 5% of the MTF; **e** Contrast measured by the uniform step wedge; **f** Noise ratio measured by the microparticle step wedges (three different sizes)

The variability components expressed as coefficients of variation (COVs) are presented in Fig. 3. For all metrics and detail sizes, inter-phantom COVs were systematically higher than intra-phantom COVs. The only exceptions were the MTF-related metrics, for which intra- and inter-phantom COVs were comparable.

Intra-, inter-, and total COVs are summarized in Table 3. For the 34 CNR metrics, the mean intra-phantom COV was 6.9% (range 1.1–14.8%), while the mean inter-phantom COV was 15.1% (range 8.4–29.4%). Intra-phantom COVs were significantly higher for lower contrast details compared to higher contrast details.

**Table 3:** Intra-phantom, inter-phantom, and total coefficients of variation (COVs) for all 64 image quality metrics evaluated in this study

Metric	Intra-phantom COV(%)	Inter-phantom COV(%)	Total COV(%)
CNR5.6 mm#1 CNR5.6 mm#2 CNR5.6 mm#3 CNR5.6 mm#4 CNR5.6 mm#5 CNR5.6 mm#6 CNR5.6 mm#7 CNR5.6 mm#8 CNR5.6 mm#9 CNR5.6 mm#10 CNR5.6 mm#11 CNR5.6 mm#12	1.10 1.45 1.83 1.85 2.27 2.78 3.67 6.44 8.13 5.22 9.68 14.19	10.22 9.90 11.23 8.85 8.43 11.56 11.85 11.33 13.30 12.01 16.14 22.94	10.28 10.00 11.38 9.05 8.75 11.91 12.43 13.20 15.69 13.17 19.12 27.30
CNR0.5 mm#1 CNR0.5 mm#2 CNR0.5 mm#3 CNR0.5 mm#4 CNR0.5 mm#5 CNR0.5 mm#6 CNR0.5 mm#7 CNR0.5 mm#8 CNR0.5 mm#9 CNR0.5 mm#10 CNR0.5 mm#11	2.01 2.12 3.41 4.97 5.91 4.84 5.90 4.19 9.78 9.96 6.75	12.19 11.98 11.69 12.82 12.84 14.23 11.31 12.97 18.71 20.43 15.38	12.37 12.18 12.21 13.79 14.24 15.07 12.82 13.69 21.30 23.07 16.99
CNR0.25 mm#1 CNR0.25 mm#2 CNR0.25 mm#3 CNR0.25 mm#4 CNR0.25 mm#5 CNR0.25 mm#6 CNR0.25 mm#7 CNR0.25 mm#8 CNR0.25 mm#9 CNR0.25 mm#10 CNR0.25 mm#11	9.97 7.35 8.19 9.42 11.33 9.87 10.32 10.28 12.24 14.83 12.71	19.67 19.80 16.08 12.48 18.16 19.00 18.20 22.43 19.51 29.38 15.73	22.30 21.25 18.06 15.77 21.54 21.47 21.07 24.96 23.02 33.27 20.38
AUMTF SpFr@50%MTF SpFr@20%MTF SpFr@10%MTF SpFr@5%MTF	5.03 0.93 1.81 6.42 9.65	5.22 1.39 2.22 6.72 11.64	7.35 1.68 2.90 9.46 15.36
Contrast#1 Contrast#2 Contrast#3 Contrast#4 Contrast#5 Contrast#6 Contrast#7 Contrast#8 Contrast#9 Contrast#10	0.01 0.02 0.02 0.03 0.05 0.06 0.10 0.15 0.31 0.62	0.04 0.56 0.82 0.74 0.23 0.39 0.51 0.64 1.04 2.57	0.05 0.56 0.82 0.74 0.24 0.39 0.52 0.65 1.09 2.65
NR328µm#1 NR328µm#2 NR328µm#3 NR328µm#4 NR328µm#5 NR234µm#1 NR234µm#2 NR234µm#3 NR234µm#4 NR234µm#5 NR125µm#1 NR125µm#2 NR125µm#3 NR125µm#4 NR125µm#5	3.72 5.49 4.55 4.29 5.64 4.06 5.96 3.71 4.33 5.01 5.63 7.21 4.37 3.87 6.17	14.23 14.23 13.55 15.24 19.15 12.52 16.24 12.49 12.50 17.92 10.94 13.96 13.46 14.96 20.06	14.76 15.33 14.36 15.85 19.98 13.22 17.39 13.09 13.27 18.63 12.40 15.80 14.20 15.49 21.05

The coefficient of variation was calculated as the standard deviation divided by the mean

*AUMTF* Area under the MTF curve, *CNR* Contrast-to-noise ratio, *MTF* Modulation transfer function, *NR* Noise ratio, *SpFreq* Spatial frequency

For the 5 MTF-related metrics, the mean intra- and inter-phantom COVs were 4.8% (range 0.9–9.7%) and 5.4% (range 1.4–11.6%), respectively.

For the 10 contrast metrics, mean intra- and inter-phantom COVs were 0.15% (range 0.01–0.62%) and 0.75% (0.04–2.57%), respectively, indicating minimal variability in contrast measurements. The 15 NR metrics showed mean intra- and inter-phantom COVs of 4.9% (range 3.7–7.2%) and 14.8% (range 10.9–20.1%), respectively, reflecting higher variability in noise-related measures.

**Fig. 4:**
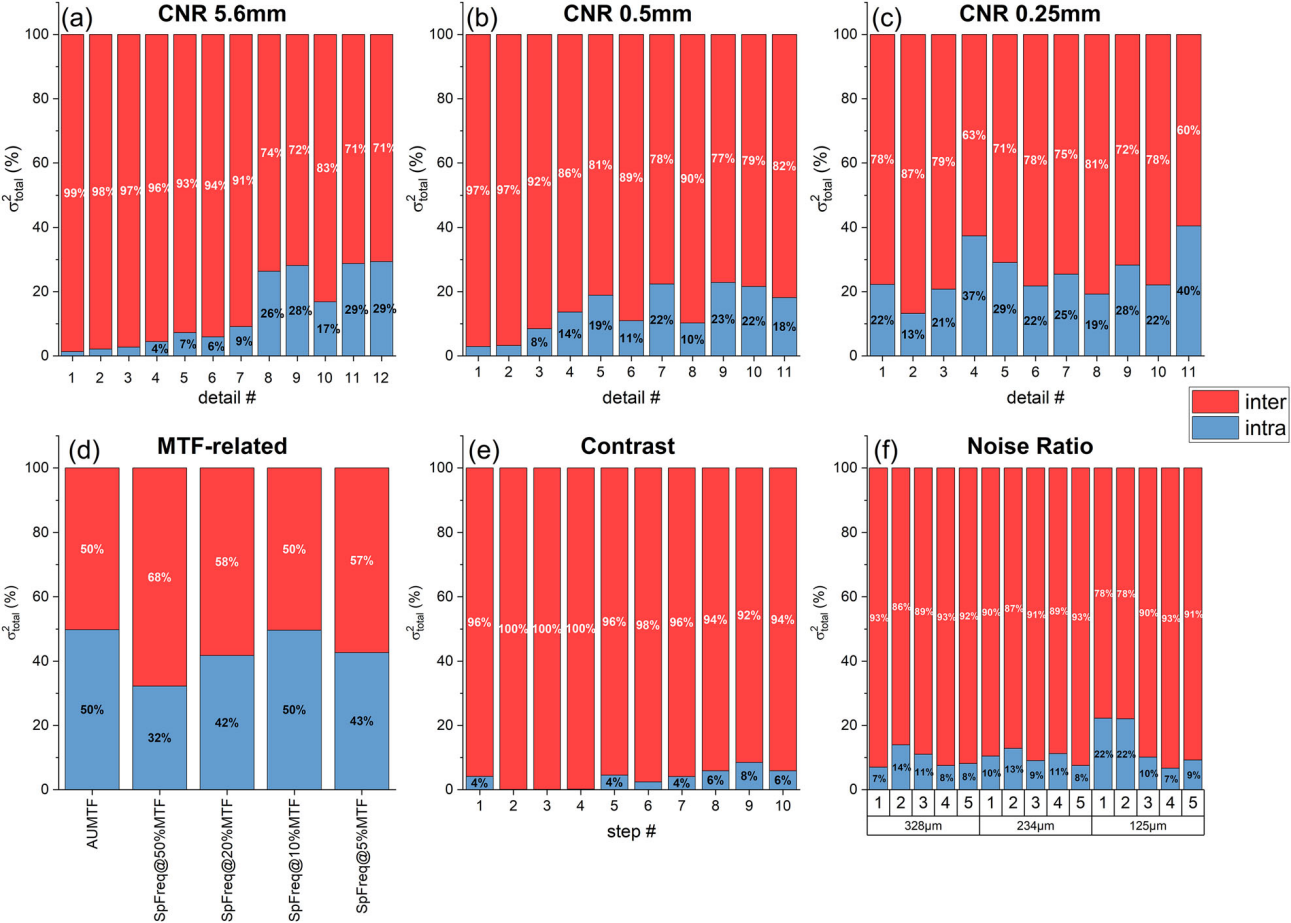
Relative contribution of intra- and inter-phantom variability to the total variability (variance) for all metrics evaluated in this study: **a** contrast-to-noise ratio (CNR) of low-contrast 5.6-mm details; **b** CNR of high-contrast 0.5-mm details; **c** CNR of high-contrast 0.25-mm details; **d** MTF-related parameters including the area under the modulation transfer function (MTF) curve (AUTF) and the spatial frequency corresponding to 50%, 20%, 10%, and 5% of the MTF; **e** Contrast measured by the uniform step wedge; **f** Noise ratio measured by the microparticle step wedges (three different sizes)

The relative contributions of intra- and inter-phantom variability to the total variance are shown in Fig. 4. For most IQ metrics, inter-phantom variability was the dominant component, contributing an average of 84.2% (range 50.3–99.9%) of the total variance. Intra-phantom variability contributed an average of 15.8% (range 0.1–49.7%).

Finally, the mean entrance dose measured was 4.655 ± 0.006 mGy (mean ± standard deviation), corresponding to a COV of 0.12%. This minimal variability confirms that the contribution of the X-ray source to the overall variability is negligible when images are acquired in manual exposure mode.

## Discussion

This study evaluated the intra- and inter-phantom variability across 64 IQ metrics derived from a standardized phantom used for mammographic quality control. The findings provide a comprehensive assessment of the reproducibility and consistency of these metrics, shedding light on sources of variability that can impact QC testing and the interpretation of mammographic performance.

Inter-phantom variability consistently exceeded intra-phantom variability for most IQ metrics, with inter-phantom contributions to total variability averaging 84.2%. This underscores the impact of differences in manufacturing, material properties, and structural orientation between phantoms. The exception was MTF-related metrics, where intra- and inter-phantom variability were similar. This is likely due to small misalignments of the bar patterns during phantom repositioning. More specifically, greater variability was observed at higher spatial frequencies (10% and 5% of MTF), where even small rotational misalignments can cause noticeable differences in MTF measurements. This highlights the sensitivity of MTF measurements to small misalignments and suggests that careful phantom handling is critical for obtaining reliable spatial resolution results.

The intra-phantom variability, expressed as coefficients of variation, was higher for CNR values of lower contrast details compared to those of higher contrast ones. This finding shows how variability is influenced by the subtlety of the measured detail and aligns with previous findings in imaging physics [18]. Lower contrast details are inherently more sensitive to noise and subtle variations in exposure conditions, making them more susceptible to variability in repeated measurements. This emphasizes that the variability of a particular metric with a particular phantom may depend not only on the metric itself but also on the characteristics of the measured detail. Such factors should be carefully considered when defining QC objectives and protocols.

The total variability (sum of intra- and inter-phantom contributions) ranged from less than 1% to over 30%, with subtle details exhibiting greater variability. This highlights two critical considerations for QC practices. First, the selection of metrics for absolute image quality assessments during equipment installation or commissioning must be chosen judiciously. Choosing inherently variable details or metrics can lead to unreliable measurements, potentially undermining the validity of the assessment process.

For instance, European guidelines recommend using the CDMAM phantom (Artinis, Nijmegen, The Netherlands) for acceptance testing of mammography systems, focusing on the threshold contrast associated with 0.1-mm diameter details [9, 20, 21]. However, recent findings by Biegała et al revealed an inter-phantom variability of approximately 11% for the threshold contrast of 0.01 mm details across nine CDMAM 4.0 phantoms analyzed using different software tools. Furthermore, they reported variability exceeding 20% when comparing different generations of CDMAM phantoms (3.4 *versus* 4.0) [22]. Similarly, Salomon et al, in developing a new phantom for digital breast tomosynthesis, observed a coefficient of variation of 32.1% among four phantoms [23]. In contrast, Davis et al, evaluated 14 Catphan phantoms for QC in computed tomography used in radiotherapy and found relatively low inter-phantom variability in metrics such as absolute HU values, uniformity, and visual assessment of low-contrast details [24].

The contradictory results reported in the literature highlight substantial differences among studies investigating inter-phantom variability. This variability is influenced by multiple factors, including the type of phantom and its manufacturing process, the specific metrics selected for image quality assessment, and the evaluation method employed. Notably, visual assessment methods are generally less sensitive to variability, whereas quantitative methods can detect even subtle differences, making them more susceptible to variability-related challenges [8, 25]. These observations underscore the importance of carefully selecting both metrics and evaluation methods to ensure reliable and reproducible results in image quality assessments.

The second consideration relates to the use of specific phantom types to compare different imaging systems under the assumption that variations in image quality metrics are solely due to differences in system performance, without accounting for potential differences between phantoms. This issue becomes even more critical in multi-system assessments across different institutions, where comparisons often rely on phantoms from different production batches or manufacturers. Such variability can introduce confounding factors that obscure true differences between mammography units, potentially leading to inconsistent conclusions regarding system performance. As demonstrated in this study and confirmed by others, inter-phantom variability can be substantial. Therefore, comparisons of device performance should, whenever possible, be performed using the same phantom or, at the very least, incorporate correction factors or normalization techniques to mitigate the impact of inter-phantom variability [26–29]. Future studies could explore standardized calibration methods to improve comparability across institutions.

Phantoms can be reliably used for testing the reproducibility of imaging systems over time without considering inter-phantom differences, particularly in reproducibility or routine QC testing, as the same phantom is consistently used in these assessments [6, 30, 31]. These scenarios focus on monitoring system variability across repeated measurements over time, where the primary source of variability is attributed to the imaging system itself. Since routine QC typically involves acquiring a single image of the same phantom at regular intervals (*e.g*., daily or weekly, depending on the QC protocol), the observed variability primarily reflects intra-system factors, with intra-phantom variability expected to be minimal. Even when routine QC tests are performed on different imaging systems, each using a different phantom, the tests can yield reliable results despite potential inter-phantom differences. This is because such differences do not affect the reproducibility of individual systems when monitored over time, underscoring the suitability of phantoms for this purpose.

Finally, phantom manufacturers could improve transparency by systematically publishing tolerances for key physical and functional properties of their phantoms. Currently, limited data are available on variations between production batches or across different brands, making it difficult to assess the impact of manufacturing variability on quality control and system performance. Standardizing and disclosing tolerances for material properties, embedded detail dimensions and contrast values would help users better interpret observed variations and facilitate more reliable inter-system comparisons. While we acknowledge that refining production tolerances may increase costs, improving transparency in phantom specifications would provide significant practical benefits to both users and quality assurance efforts.

This study evaluated inter-phantom variability using only the 10-mm test plate at a high dose, optimizing the visibility of subtle details allowing the measurement of all 64 metrics. This approach differs from standard QC conditions where the test plate is combined with a 35-mm PMMA stack, and exposure is guided by automatic exposure control. While such conditions align with clinical protocols, they would significantly attenuate the phantom details, reducing the number of measurable metrics and limiting contrast visibility. The decision to use the high-dose, 10-mm test plate was made to maximize detail visibility, ensuring a comprehensive evaluation of all image quality metrics.

Two of the original 24 phantoms were excluded from the analysis due to being identified as outliers based on one of their CNR values. One detail of these two phantoms, TM000580 and TM000593, displayed anomalous performance that deviated significantly from the other phantoms. Although the definition of what constitutes a “defective” phantom is somewhat arbitrary, once established, it must be consistently applied. Specifically, the apparent “swapped” CNR values for the first two 0.5-mm details of phantom TM000580 represent a defect rather than a mere instance of variability. The exclusion of these phantoms helped to ensure the integrity of the analysis, as including them would have introduced skewed data that could distort the observed variability. Therefore, the results presented here reflect the remaining 22 phantoms, which exhibited more consistent and reliable performance, offering a more accurate assessment of intra- and inter-phantom variability.

In conclusion, our analysis of 64 IQ metrics across 22 phantoms from the same manufacturer showed an intra-phantom variability ranging from 0.14 to 6.9% and an inter-phantom variability ranging from 0.75 to 15.1%. Inter-phantom variability contributed 84.2% to total variability, highlighting its dominance.

These findings emphasize the importance of using the same phantom for inter-system comparisons to avoid confounding results. Conversely, phantoms are well-suited for assessing system reproducibility over time, focus on inter-system variability while consistently using a single phantom.

## Data Availability

The datasets analyzed during the current study are available in the Zenodo repository, 10.5281/zenodo.14363482.

## Abbreviations

AUMTF: Area under the MTF curve
AutoPIA: Automatic phantom image analysis
CNR: Contrast-to-noise ratio
COV: Coefficient of variation
IQ: Image quality
MTF: Modulation transfer function
NR: Noise ratio
QC: Quality control
ROI: Region of interest
